# Stimuli-Responsive Polypeptides for Biomedical Applications

**DOI:** 10.3390/polym10080830

**Published:** 2018-07-27

**Authors:** DaeYong Lee, N. Sanoj Rejinold, Seong Dong Jeong, Yeu-Chun Kim

**Affiliations:** Department of Chemical and Biomolecular Engineering, Korea Advanced Institute of Science and Technology (KAIST), Daejeon 34141, Korea; winleeok@kaist.ac.kr (D.L.); sanojrejinold@kaist.ac.kr (N.S.R.); sdjeong@kaist.ac.kr (S.D.J.)

**Keywords:** stimuli-responsiveness, polypeptides-based materials, drug and gene delivery systems

## Abstract

Stimuli-responsive polypeptides have gained attention because desirable bioactive properties can be easily imparted to them while keeping their biocompatibility and biodegradability intact. In this review, we summarize the most recent advances in various stimuli-responsive polypeptides (pH, reduction, oxidation, glucose, adenosine triphosphate (ATP), and enzyme) over the past five years. Various synthetic strategies exploited for advanced polypeptide-based materials are introduced, and their applicability in biomedical fields is discussed. The recent polypeptides imparted with new stimuli-responsiveness and their novel chemical and physical properties are explained in this review.

## 1. Introduction

Stimuli-responsive polymers can respond to specific stimuli such as pH, temperature, redox-potential, light, enzymes, etc. and change their chemical or physical properties [1]. Stimuli-triggered changes by a specific stimulus elicit the desirable properties only at the diseased sites while minimizing unwanted side effects [1,2]. Considering their controllable characteristics, stimuli-responsive polymers have been widely used in various biomedical fields [1,2]. Among the polymers, polypeptide-based materials have recently been in the limelight because polypeptides possess innate biocompatibility and degradability and numerous biological functions which are determined by their unique secondary protein conformations, random coils, β-pleated sheets and α-helices [3,4].

Generally, polypeptides are prepared by two synthetic methods, solid phase peptide synthesis (SPPS) and *N*-carboxyanhydride (NCA) ring-opening polymerization [5]. The former one is the conventional method that is able to synthesize peptides by sequencing amino acid residues [6]. However, it is difficult for SPPS to elongate the peptide backbone due to extremely low yields and non-cost-effectiveness [6]. The latter one is the prevalent method that is capable of synthesizing elongated polypeptides and forming various architectures although sequencing amino acid residues is impossible [5]. Moreover, using the living polymerization methods, the chain length of polypeptides or the type of polymers can be controlled [7,8].

Stimuli-responsive polypeptides can be simply synthesized with naturally occurring amino acid residues without complicated modifications in that some functional group-bearing amino acids, such as cysteine, glutamic acid, aspartic acid, histidine and methionine, possess an innate stimuli-responsive characteristic [7,8]. To impart sophisticated functionality to polypeptides, the desirable building blocks are attached to the side chains of the polypeptides using simple conjugating methods [9]. Therefore, stimuli-responsive polypeptides are easily customized by the introduction of a desirable functional moiety responding to a specific stimulus.

**Figure 1:**
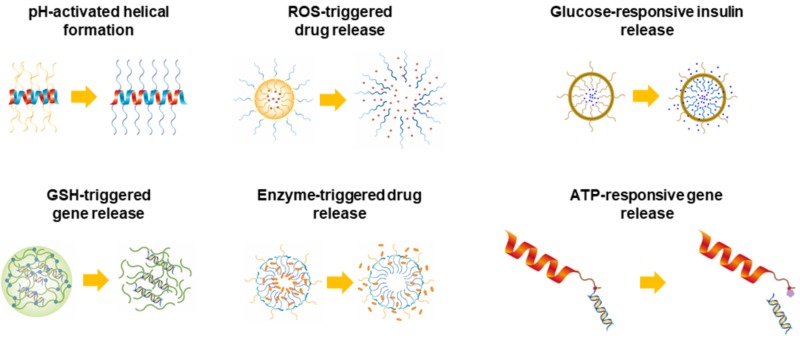
Schematic illustration of representative stimuli-responsive polypeptides used in biomedical fields.

In this review, we discuss the recent progress in stimuli-responsive polypeptide systems including pH, reduction, oxidation, glucose, adenosine triphosphate (ATP), and enzymes and elucidate the theoretical approaches for the past five years (Figure 1). For pH-responsive polypeptides, although they are prevalently exploited, several polypeptides possessing the property of conformational transitions via pH are mainly introduced in this review, which were very recently developed, and the future direction for the use of pH-responsiveness is suggested as well in this review (Figure 1) [10,11,12]. Additionally, the new stimuli-responsive characteristics, oxidation- and ATP-responsiveness, are covered in this review (Figure 1). More emphasis is given to the various biomedical applications that use stimuli-responsive polypeptides in this review.

## 2. Synthetic Strategies

As mentioned in the introduction part, the synthetic methods of polypeptides breaks down to two methods, SPPS and NCA polymerization. Those two methods possess obvious advantages and innate drawbacks at the same time. When it comes to the SPPS method, the amino acid residues, which elicit the sophisticated bioactive functions depending on the purpose, can be sequenced [6,13]. For instance, cell-penetrating peptides, widely used in biomedical applications [14], can be synthesized employing this synthetic method. In addition, peptide sequences recognized by enzymes are elaborately synthesized. When it comes to the synthetic method, the amino acid residue is first attached to polystyrene beads that are functionalized on the surface [6,13]. Thereafter, the protecting group usually attached to the amine of the amino acids is removed before amino acid residues are conjugated to the amino acid residue already bonded to the beads in regular sequence [6,13]. In the SPPS method, the amide formation is proceeded using carbodiimide chemistry, and deprotecting methods are determined depending on the types of protection groups [6,13]. However, innate drawbacks such as high production cost, low yield, and difficulty in elongation of the chain length are seen in the SPPS method. 

NCA polymerization for polypeptide synthesis has drawn attention in biomedical fields due to its feasibility for chain elongation, low production cost, and high yield, even though sequencing the amino acid residues is impossible [5]. NCA monomers characterized with a five-membered ring structure are usually prepared by the Leuchs or Fuchs-Farthing method [5]. In the case of functional group-bearing amino acids such glutamate, lysine, arginine, etc., the moiety is capped by the protecting group and selective cleaved under specific conditions to prevent unwanted side reactions. The cyclic structure is vulnerable to nucleophiles (primary amines) acting as an initiator because NCA is kinetically favorable but thermodynamically unstable. The nucleophile attacks the carbonyl group of the NCA, resulting in ring-opening. After the ring-opening of the NCA, the amine moiety exposed by CO_2_ generation re-attacks another NCA monomer. Based on this synthesis, polypeptides can easily be synthesized. Although sequenced polypeptides cannot be fabricated with the NCA polymerization, the regulation of its molecular weight and the high productivity are advantageous Moreover, this novel living NCA polymerization method was developed to achieve a narrow molecular weight distribution and elaborate control of the molecular weight [7]. Hexamethyldisilazane (HMDS) used as a living initiator can open the ring structure and drive polymerization. The driving force of the controlled polymerization is that the trimethylsilyl (TMS) carbamate group only reacts with another NCA monomer by TMS transfer. Using this method, various polypeptide architectures have been achieved [8,15,16]. 

Generally, the synthetic method for artificial polypeptides has been intensively employed in drug or gene delivery systems. For drug delivery systems, block copolypeptides possessing amphiphilicity are synthesized to simultaneously provide drug encapsulation and solubilization in water [7,8]. On the other hand, cationic polypeptides containing amines or guanidine have been developed for gene delivery systems to condense genes, and to interact with lipid plasma membranes [17]. When it comes to recent research trends on polypeptides, the regulation of secondary protein conformation has been of an interest in biomedical fields [3,4]. Among three prevalent secondary structures, the alpha helical conformation has been intensively investigated for enhanced cell-penetrating agents [18]. To synthesize artificial helical polypeptides, the side chain of polypeptides were elongated by conjugating sterically unhindered hydrocarbons [18]. The elongated side chains renders the polypeptide to be spirally folded causing hydrophobic interactions within the side chains which are dominant to electrostatic repulsion [18,19]. Based on the finding, several artificial helical polypeptides have been developed by using cationic moieties at the peripheral side chain. In terms of the cell-penetrating effectiveness, artificial helical polypeptides considerably surpass conventional cell-penetrating peptides (CPP) such as TAT, pVEC, etc. [19,20,21,22]. 

Depending on the use of synthetic strategies, polypeptides are easily customized in various biomedical fields. By using recently devised synthetic strategies, various polypeptide-based materials endowed with stimuli-responsiveness have been developed to compensate for drawbacks and to improve their applicability. Based on the synthesis, recent stimuli-responsive polypeptides are discussed in this review. 

## 3. pH-Responsive Polypeptides

pH-responsive polypeptides have been the most intensively investigated ones in drug and gene delivery systems because pH variations are widely observed in biological systems, intracellular compartments (lysosomes and endosomes), specific organs (gastrointestinal tract and vagina), and diseased sites (cancer) [2]. Recently, conformation-transformable polypeptides (CTP) via pH variations were developed to impart specificity at the diseased site. The polypeptides were converted from low helicity or random coils to an intact helical conformation at low pH, thereby possessing a membrane-disrupting capability. The characteristic of pH-triggered conformation transitions was exploited in cancer targeting and therapy systems. 

**Figure 2:**
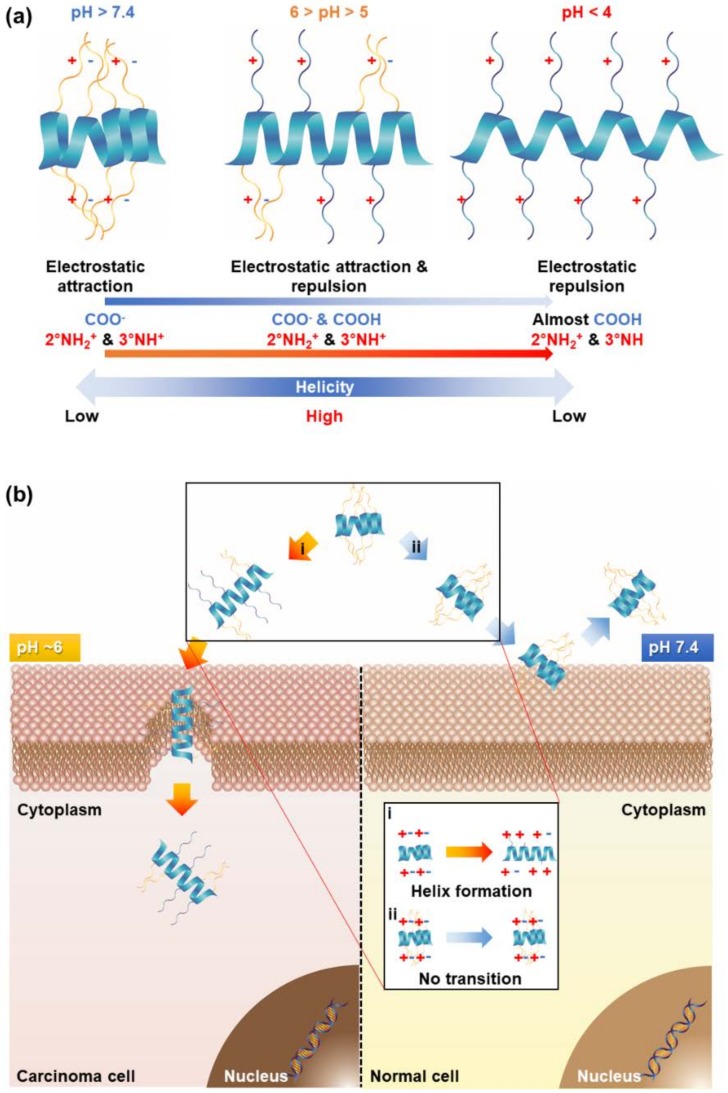
Design of pH-controllable cell-penetrating polypeptides (PCCPs), and proposed mechanism of pH-controllable helicity and selective cellular penetration. (**a**) Proposed mechanism of PCCP possessing a pH-activated cell-penetrating property exclusively at the tumor extracellular matrix. 2° and 3° indicate “secondary” and “tertiary”, respectively. (**b**) Schematic illustration of the PCCP undergoing pH-dependent conformational transition induced by the charge balances of two opposite ions. Reprinted permission from [11]. Copyright 2017, Elsevier.

Generally, the tumor extracellular matrix is somewhat acidified by the overproduction of lactate from glycolysis (pH ~ 6) [23]. Therefore, the introduction of pH-responsiveness to polypeptides provides selectivity to cancer sites. Previous studies have suggested a new approach for specific cancer targeting by grafting a pH-responsive property onto a polypeptide (Figure 2) [11].

The polypeptides underwent pH-responsive conformation transitions whereby the electrostatic interactions within the side chains were changed [11]. A low helical propensity was exhibited at physiological pH because the electrostatic attractions were dominant [11]. However, an intact helix was formed at a low pH (5 < pH < 6) due to the balance of electrostatic attractions and repulsions [11]. Using this characteristic, the pH-responsive polypeptide was capable of selectively penetrating the cancer cells at low pH [11]. In this study, they differentiated three different cell-penetrating polypeptides to compare a pH-triggered cell-penetrating characteristic: non-transition, beta-to-low helix, and low-to-high helix [11]. 

**Figure 3:**
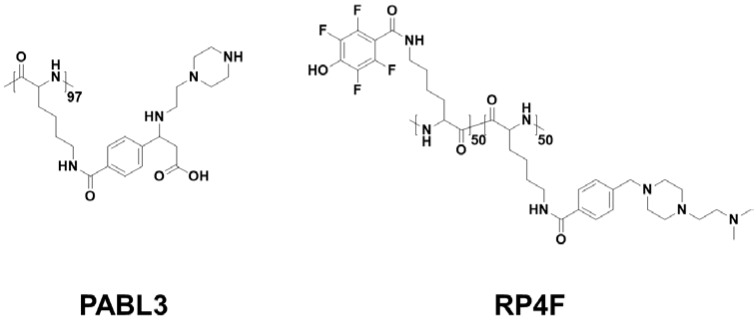
Representative pH-responsive polypeptides undergoing the conformational transition.

**Figure 4:**
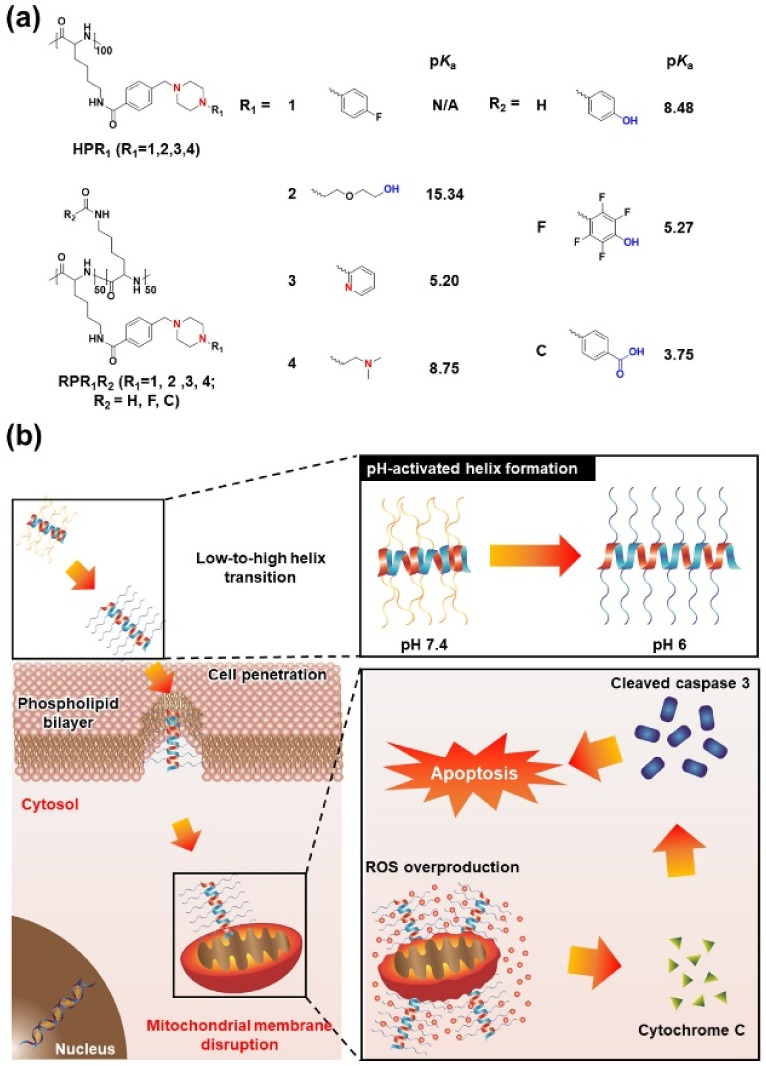
Schematic illustration of several conformational transitions and the selective pro-apoptotic mechanism. (**a**) Chemical structures and p*K*_a_ values of the homo cationic helical polypeptides and random cationic helical copolypeptides. (**b**) pH-activated mitochondria-destabilizing helical polypeptides selectively translocates across carcinoma plasma membranes and then, aggravates the disruption of mitochondria membranes thereby inducing pro-apoptosis. All the p*K*_a_ values were estimated by Marvin and JChem calculator plugins. Reprinted permission from [12]. Copyright 2017, Elsevier.

Among them, PABL3, a pH-triggered low-to-high helix, showed much higher calcein delivery effectiveness and also a pH-activated cell-penetrating property at pH 6, indicating that the pH-responsive helical formation contributed to selective cancer targeting [11] (Figure 3). For an advanced version of pH-responsive transformable polypeptides, they developed a novel polypeptide which was able to simultaneously achieve cancer targeting and therapy (Figure 3 and Figure 4) [12]. Likewise, the pH-responsive polypeptide RP4F was converted to a high helical formation at tumor pH because the electrostatic attractions within the side chains were diminished by the protonation of the anion-donating moieties (Figure 4a) [12]. Therefore, the pH-activated helical formation enabled the polypeptides to selectively penetrate the cancer cell membranes (Figure 4b) [12]. When RP4F was transformed to the high helix at low pH, the anion-donating groups became hydrophobic due to protonation, thereby forming the cationic amphipathic helix that can target and disrupt mitochondrial membranes (Figure 3 and Figure 4b) [12]. The pH-activated helix targeted and then destabilized mitochondrial outer membranes, which created oxidative conditions [12]. The overproduced reactive oxygen species activated apoptosis signaling, thereby inducing apoptosis (Figure 4b) [12]. It was demonstrated that RP4F specifically targeted cancer sites and suppressed tumor proliferation in vivo [12].

The characteristic of conformational transition via pH-responsiveness was also exploited as novel antimicrobial agents [10]. They include glutamic acid which has an important role in pH-triggered coil-to-helix transition [10]. At physiological pH, the random coil conformation is present by the generation of electrostatic attractions within the side chains [10]. In contrast, the polypeptide rapidly switches to a helical structure due to the protonation of the carboxylic acid groups [10]. Using the physical property, the pH-activated helix selectively inhibited the rapid growth of *Helicobacter pylori* (*H. pylori*) in the stomach [10]. They verified that the pH-activated helical polypeptide specifically killed *H. pylori* only without damaging normal cells or commensal bacteria via in vivo model [10]. Compared to the combination therapy using commercialized drugs, the therapeutic efficiency of the polypeptide was remarkably comparable to that of the conventional treatment while the side effects were highly minimized because the polypeptide became completely inactive outside of the stomach [10]. 

Additionally, CTP was utilized as an endosome-escaping agent by pH-sensitive hydrogen bonding [24]. The hydrogen bonding pattern of 1,2,3-triazole moiety located at the middle of the side chains was changed from binary to unitary in acidic conditions because the nitrogen in the triazole was protonated, thereby forming the intact helical conformation at a low pH through the conversion of hydrogen bonding patterns within the side chains [24]. In detail, the hydrogen of the 1,2,3-triazole had an interaction with that of amide bonds in the polypeptide backbone at neutral pH, which resulted in the disruption of the helical formation [24]. Contrastively, the hydrogen donating ability was weakened by the protonation of the 1,2,3-triazole groups, giving rise to the intact helical formation [24]. The pH-activated helical conformation strongly destabilized the plasma lipid membranes and then accelerated the escape of endosomes [24].

Another strategy for endosomal escape was that beta sheet-to-helix transformable peptides controllably release encapsulated drugs [25]. At physiological pH, the peptide exhibited a beta-sheet structure while the secondary conformation was converted to a helix at lysosomal pH, thereby escaping from lysosomes [25]. Furthermore, the morphology of nanoparticles were also changed with pH variation from sphere to fiber [25]. When the nanospheres were transformed to nanofibers, there was a considerable release of the encapsulated drug because the electrostatic repulsions were generated between doxorubicin and the primary amine groups [25]. It was shown that CTP-based nanospheres possessed a high tumor-inhibiting capability in vitro and even in vivo [25].

Although this approach was not applied in biomedical fields, its applicability as a pH-responsive CTP was shown by introducing (4*S*)-aminoproline in the peptide chain [26]. Generally, the secondary conformation of polyproline imbues a triple helix because of ring strain [26]. It was shown that polyproline containing (4*S*)-aminoproline residues was changed to a random-coiled conformation under acidic conditions [26]. The driving force of the pH-triggered conformational change was that the protonated amine interacted with the adjacent carbonyl group, forming a boat conformation [26]. These findings show the possibility for developing pH-sensitive collagen-based materials which can selectively release cargo molecules at low pH [26]. A similar concept was reported by imidazoylated poly-l-lysine (PLL) [27]. PLL is endowed with a random-coiled conformation because of dominant electrostatic repulsion. To form a pH-sensitive helical structure, the imidazole moieties were attached to the primary amines of the PLL by the formation of amide bonds [27]. It was observed that imidazoylated PLL underwent a pH-triggered conformational transition from an alpha helix to a random coil structure with a decreasing pH level [27]. The reason for the pH-triggered random coil formation was that the imidazole rings were protonated at acidic pH [27]. This concept of a conformation-transformable characteristic by pH was used in a pH-triggered drug release system at a specific target site [27]. 

The recently devised pH-responsive polypeptides undergo a conformational transition [10,11,12,25,27]. In general, cationic helices have strong electrostatic attractions with the lipid plasma membranes and then disrupt or distort the structure of lipid plasma membranes [10,11,12]. To be active at the desirable site, pH-responsive groups were introduced in the side chain or backbone of the polypeptide, undergoing pH-triggered conformational transitions from low to high helicity [10,11,12]. For this strategy, the selectivity and effectiveness were considerably improved while minimizing the undesirable effects. Therefore, the pH-responsive CTPs are promising materials for selective targeting and therapy.

## 4. Reduction-Responsive Polypeptides

The responsiveness of reduction has been intensively exploited in intracellular delivery systems because the redox potentials are highly polarized between intracellular and extracellular environments [28,29]. When it comes to intracellular conditions, glutathione (GSH), a bio-reducing agent, is highly produced to prevent oxidative stress (100-to-1000 fold higher than extracellular conditions), which is a specific stimulus in only intracellular surroundings [28,29]. The common strategy is to impart disulfide bonds in polypeptide systems to deconstruct the delivery carrier at the target site because the disulfide bonds can be reduced by GSH [30]. The segments of polypeptide are cross-linked by the formation of disulfide bonds, achieving a network structure that can tightly capture genes or drugs by electrostatic attractions or hydrophobic interactions, respectively [31,32,33]. Consequently, a network carrier linked with disulfide bridges is suitable for intracellular delivery.

**Figure 5:**
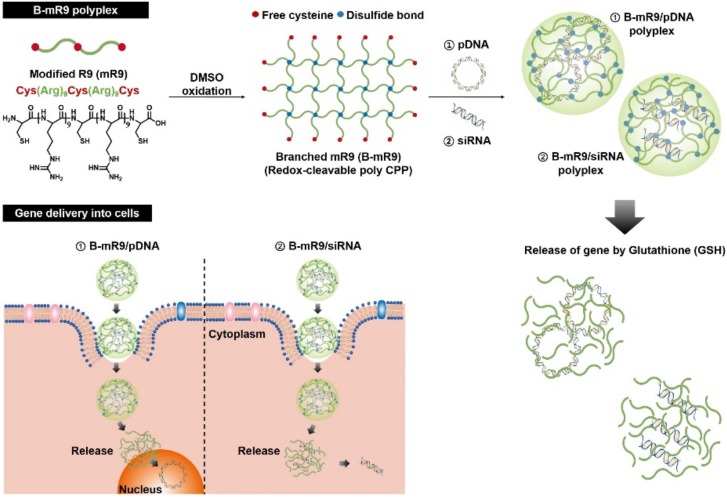
Schematic illustration of the synthesis of the branched-modified R9 (B-mR9) cell-penetrating peptide (CPP) and construction of pDNA and siRNA polyplexes. Positively charged B-mR9 is constructed with negatively charged genes through electrostatic interactions. B-mR9 polyplexes is delivered into cells by means of the permeability of the CPP. The branched structures of B-mR9 can then be cleaved by the reductive conditions of the intracellular matrix releasing the pDNA or siRNA into the nucleus or cytoplasm, respectively. Reprinted with permission from [30]. Copyright 2017, Elsevier.

In the case of gene delivery systems, various cationic polypeptides have been widely used as a gene-condensing agent although the polypeptides are incapable of readily releasing the encapsulated genes at the target site due to a strong electron charge density [30]. To overcome this hurdle, oligo cationic polypeptides were cross-linked by disulfide bond formation, which provides an enhanced cationic charge density and a GSH-triggered gene release at the same time [30]. Only nona-arginine (R9) used as a CPP is insufficient to condense huge gene molecules due to the extremely low cationic charge density [30]. Using the modified R9 which included three thiol groups at the ends and middle of the R9 segments, the branched structure was easily fabricated by forming disulfide bonds (Figure 5) [30]. The GSH-cleaved branched structure remarkably improved the gene-condensing capability and effectively released genes in the cytosol via deconstruction of the branched structure (Figure 5) [30]. In comparison to R9, the branched-modified R9 possesses a superior capturing and GSH-triggered gene release ability and furthermore, had even higher gene transfection and silencing efficiencies in the absence of cytotoxicity [30]. Even in a mouse study, the branched-modified R9 inhibited tumor proliferation by delivering small-interfering RNA (siRNA); thus, it was used as an effective gene-delivering agent [30].

Another study also introduced disulfide bridges into the artificial helical polypeptide containing thiol groups [31]. Thiol groups were conjugated to the outskirt of the side chains to form a disulfide bond-mediated cross-linked structure [31]. The cross-linked structure tightly captured siRNA, which protects it against enzymatic degradation, and then exposed the siRNA in the cytoplasm by the reduction of the disulfide bonds [31]. The cross-linked helical polypeptide strongly condensed the siRNA and translocated the siRNA into the cytoplasm compared to the uncross-linked helical polypeptide, indicating that the cross-linked structure considerably elevated the cationic charge density [31]. Moreover, a higher uptake efficiency of the cross-linked helical polypeptide was shown with low cytotoxicity compared to lipofectamine 2000 which is a commercial transfection agent [31].

The utilization of disulfide bridges has also been found in drug delivery systems [34]. The block copolypeptide composed of polyethylene glycol (PEG) and poly(aspartic acid-*random*-tyrosine) was cross-linked with disulfide bonds to effectively encapsulate drugs [34]. The cross-linked nanoparticles had a relatively lower hydrodynamic diameter and attained on-off drug release via GSH-responsiveness [34]. For tight encapsulation drugs in the core of the nanoparticles, the therapeutic effectiveness of the cross-linked nanoparticles were remarkably higher in vivo compared with the other groups [34]. Another strategy of disulfide bond-mediated cross-linking is to include cysteine residues on the block copolypeptide [33]. A block copolypeptide contained PEG as a hydrophilic segment and poly(cysteine-*random*-phenylalanine) loading the drugs. The cysteine residues were oxidized to form the cross-linked structure, resulting in the formation of compact nanoparticles [33]. The tight nanoparticles selectively responded to intracellular conditions via GSH and then, released the encapsulated drugs in the cytosol [33]. Although disulfide bonds have been widely exploited as a cross-linking agent, a bio-reducible bond is able to act as a linker between hydrophilic and hydrophobic segments [32]. A PEG block was attached to the end of a hydrophobic polypeptide using a disulfide bond [32]. Polypeptide micelles were passively internalized into the cells and deconstructed by the evacuation of the PEG blocks from the micelles, thereby releasing the cargo molecules [32]. The occurrence of GSH-triggered dissociation accelerates the prompt release of encapsulated drugs into the cytoplasm [32].

Various approaches utilizing the reduction-responsive characteristic have mainly focused on intracellular delivery systems due to the overproduction of GSH [32,33,34]. The introduction of disulfide linkages in drug or gene delivery carriers has enabled nanoparticles or nanocomplexes to be tightly compacted for both enhanced particle stability and controlled release at the target site [32,33,34]. However, in addition to the disulfide bridge, other GSH-responsive groups should be introduced in the polypeptide systems to enhance the GSH-sensitivity.

## 5. Oxidation-Responsive Polypeptides

Reactive oxygen species (ROS) that are highly reactive are produced as a natural byproduct of cell metabolism and also mediate cell signaling and homeostasis [35]. In general, the intracellular ROS level in cancer cells is excessively elevated to activate proliferation, angiogenesis, and metastasis [35,36]. Recently, oxidation-responsive characteristics have been intensively investigated in drug delivery and pro-drug systems as a new promising stimulus [37]. Like reduction-responsive systems, ROS-responsive systems have been mainly used for selective intracellular delivery due to the considerable difference in ROS concentration between the tumor extracellular and intracellular conditions [38].

**Figure 6:**
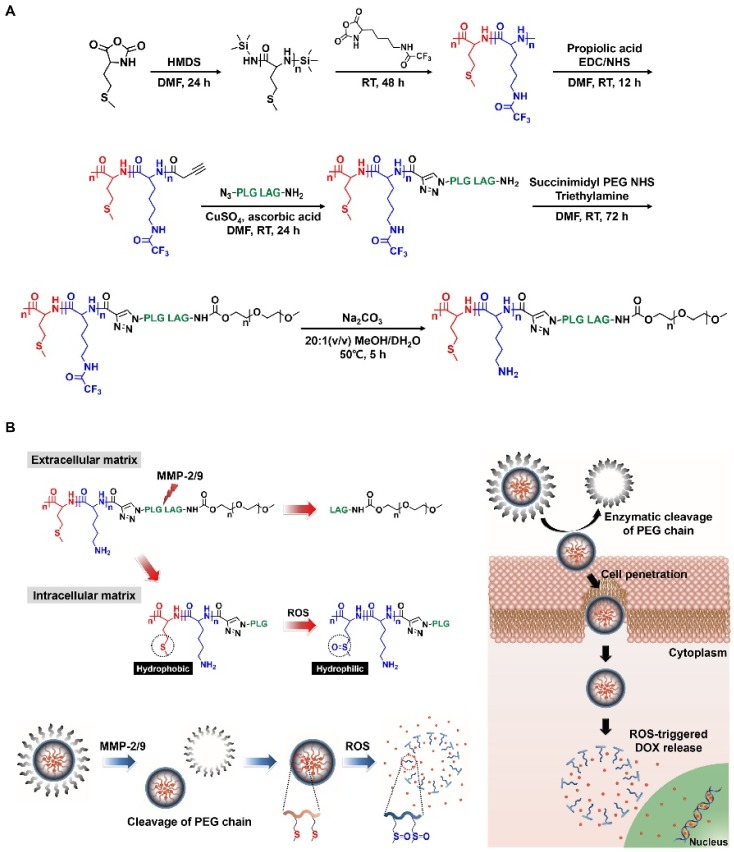
Synthesis procedure and schematic illustration. (**A**) Synthesis of poly(l-methionine-*block*-l-lysine)-PLGLAG-PEG (MLMP). (**B**) Schematic illustration of the anticancer drug delivery procedure. The MMP-sensitive linker of MLMP can be cleaved in the extracellular matrix of cancer cells. After enzymatic cleavage of the PEG chains, poly-l-lysine chains are revealed and can penetrate cells because of its CPP property. The hydrophobic thioether groups of the poly-l-methionine chains are then converted to hydrophilic sulfoxide groups by the intracellular matrix rich in ROS. Through the conversions of the polypeptide chain, the micelle structures of the MLMP (DOX) are destroyed, and then, the encapsulated DOX is released into the cells. Reprinted with permission from [39]. Copyright 2017, Elsevier.

A thioether moiety that is vulnerable to oxidation exhibits a ROS-triggered phase transition by oxidizing thioether to sulfoxide or sulfone [37,39]. When thioether is oxidized, its physical property is significantly changed from hydrophobic to hydrophilic [37,39]. To graft thioether onto drug delivery systems, methionine, which is a thioether-containing essential amino acid, was copolymerized with lysine, thereby synthesizing poly(methionine-*block*-lysine) [39]. Block copolypeptides imparted with ROS-responsiveness underwent a phase transition with increasing ROS levels in which the polymethionine blocks became hydrophilic via thioether oxidation, which resulted in the deconstruction of its micelle structure (Figure 6) [39]. As a result, the collapsed nanoparticles selectively released the drugs (Figure 6) [39]. The block copolypeptides specifically released the drug in ROS-overproducing cancer cells because the cell viability was much lower so that the polymethionine blocks consequently were oxidized, and converted to hydrophilic segments [39]. Another strategy was to impart thioether groups artificially into the side chain of polypeptides to imbue a ROS-responsiveness [40]. Using a thiol-ene reaction, two thioether groups were attached to the side chain of poly(γ-propargyl-l-glutamate) [40]. The high helical propensity of the polypeptide was exhibited under ROS-deficient conditions [40]. However, the helicity was reduced when the thioethers were oxidized under ROS-rich conditions, indicating that the conversion to sulfone destabilized the formation of helices [40]. Additional research using a thioether as a phase-transforming group was that the building block, which simultaneously included ROS- and hypoxia-responsive properties, was attached to the serine residue of a PEG-polyserine block copolymer [41]. They prepared two different block copolymers: photosensitizer-conjugated and thioether-including polymers [41]. The accumulated nanoparticles in the tumor was activated by light irradiation, giving rise to ROS generation [41]. The elevated ROS concentration promoted the oxidation of the thioether moieties, thereby releasing the encapsulated tirapazamine (TPZ) [41] TPZ is activated by highly reactive radical species in hypoxic environments, thereby stimulating apoptotic pathways [41].The ROS-responsive nanoparticles suppressed tumor proliferation in vivo by oxidative stress [41].

Using a cysteine residue, a new thioether-bearing polypeptide was developed by a S_N_2 reaction [42]. A cholesterol moiety was conjugated to the end of the thiol in the cysteine by preserving the thioether [42]. Using PEG-amine as a macro initiator, cholesterol-conjugated cysteines were polymerized to form ROS-responsive micelles [42]. With increasing ROS levels, the micelles were converted into vesicle structures in which the alpha helix was transformed to a beta sheet by the oxidation of thioether [42]. Through the ROS-triggered morphological change, the encapsulated drugs were able to be selectively delivered to the target sites.

In addition, the same ROS- and hypoxia-responsive polypeptides were used in controlled insulin delivery systems with the help of a microneedle. Insulin and glucose oxidase were co-loaded in block copolypeptide-based nanoparticles [43]. When glucose binds to glucose oxidase, a glucose molecule is converted to gluconic acid and hydrogen peroxide, thereby fostering acidic and ROS-rich conditions at the same time [43]. The block copolypeptide loses the hydrophobic block due to the oxidation of the thioether groups, thereby selectively releasing insulin [43].

A phenylboronic acid moiety also has ROS-sensitivity because the boronic acid is dissociated from the phenylboronic acid via ROS [35]. The general approach is that *p*-hydroxymethylphenylboronic acid (PHMBA) groups are attached to the peripheral side chains of a polypeptide by forming an ester, carbonate or carbamate bond [44]. When these linkers are faced with ROS, boronic acid is dissociated from the linker, and then, the linker is completely shattered by forming carbon dioxide and Quinone methide. A recent study reported that the ROS-cleavable linker was exploited as a moiety providing hydrophobic interactions and a glutathione-depleting agent [45]. PHMBA was linked to the side chain of the polypeptide with a carbonate bond [45]. When exposed to the intracellular environment, the polypeptide-based nanoparticles released glucose oxidase and Quinone methide by the ROS-responsive dissociation of the linker, which resulted in amplified oxidative stress to cancer cells [45]. An elevated ROS level and depleted GSH highly induced apoptosis [45].

**Figure 7:**
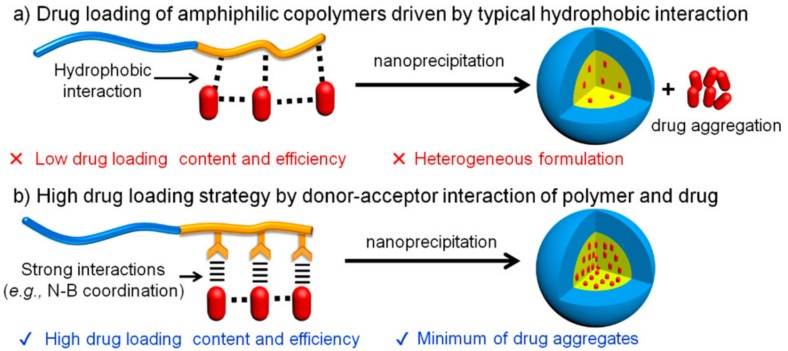
(**a**) Unwanted formation of drug aggregates during drug-loading via hydrophobic interactions. (**b**) Quantitative and high drug-loading enabled via specific drug–polymer coordination interactions. Reprinted with permission from [46]. Copyright 2018, American Chemical Society.

Another report was that a PHMBA moiety can enhance the drug-loading efficiency and the ROS-responsive release of encapsulated drugs (Figure 7) [45]. The PHMBA moiety linked to the side chain of glutamate residues with an ester had pi-pi interactions with the chemotherapeutic drugs and accepted the non-pair electrons of the drugs, thereby achieving a high drug-loading efficacy (Figure 7) [46]. With increasing ROS levels, the loaded drugs were released in which the PHMBA moiety was highly cleaved [46].

The use of PHMBA-containing ROS-cleavable linkers were also found in controlled insulin delivery systems [47]. The PHMBA moiety was linked to a PEG-polyserine block copolypeptide by the formation of carbonate bonds [47]. The insulin and glucose oxidase were co-loaded in nanovesicles, and then, using microneedles, the loaded insulin was administrated into the systemic circulation [47]. In a hyperglycemic state, glucose oxidase significantly converts glucose to gluconic acid and hydrogen peroxide, which result in creating ROS-rich environments [47]. Consequently, the PHMBA-bearing linkers are dissociated, thereby providing controlled insulin release by the loss of the hydrophobic domains [47].

ROS-responsive polypeptides mainly narrow down to two types: thioether- [39,40,41] or PHMBA-bearing polypeptides [45,46,47]. In the case of thioether-bearing polypeptides, a thioether moiety, which is vulnerable to oxidation, enables the polypeptide to convert its phase. When it comes to drug delivery systems, stimuli-triggered phase transition plays an important role in specific delivery to the target site. For this reason, thioether-bearing polypeptides provide ROS-triggered cargo delivery to the desirable site, resulting in high therapeutic effectiveness with low unwanted cytotoxicity. As far as PHMBA-containing polypeptides are concerned, PHMBA is covalently bonded to the outskirt of the polypeptide side chains with ester, carbonate, or carbamate. The PHMBA-bearing linker that responds to ROS is rapidly cleaved to carbon dioxide and Quinone methide, which brings about the loss of the hydrophobic domain but the release of a GSH-depleting agent. Therefore, PHMBA-including linkers have been grafted onto drug delivery systems to amplify the oxidative stress in cancer cells. This strategy provides a synergistic therapeutic platform for cancer therapy.

## 6. Glucose-Responsive Polypeptides

Glucose-responsive characteristics have been widely applied in controlled insulin delivery systems because insulin should be released at a high blood glucose level [48]. To impart glucose-responsiveness into a polypeptide, a phenylboronic acid (PBA) moiety was conjugated to the side chain of a polypeptide using carbodiimide chemistry [49]. PBA and its derivatives have a strong affinity with 1,2-diol compounds by forming a 5- or 6-membered ring structure that is thermodynamically and kinetically favorable [48,49]. When a glucose molecule binds to the PBA, the boron is negatively charged, making it water soluble [49]. However, naked PBA cannot bind 1,2-diol compounds at physiological pH because its *p*K_a_ value is too high to form the ring structure [49]. Therefore, an electron-withdrawing group (EWG) was attached to the benzene ring of PBA to reduce the electron density of boron [49]. EWG-tagged PBA moieties can dominantly capture glucose molecules even at neutral pH, thereby the phase transition occurs [49].

The general approach for the synthesis of glucose-responsive polypeptides has been to conjugate 3-aminophenylboronic acid to the carboxylic acid groups of poly-l-glutamic acid or poly-l-aspartic acid. Using poly-l-glutamate, a glucose-responsive polypeptide was successfully synthesized using carbodiimide chemistry [49]. In that study, glucose-responsive polypeptides used as a glucose-triggered phase-transforming block were attached to the side chain of sodium alginate to form a glucose-responsive hydrogel [49]. At a normal glucose level, the glucose-responsive nanogel rarely released insulin, and its size was kept consistent because the glucose-responsive polypeptide segments were hydrophobic [49]. However, when the glucose level became elevated, the glucose-responsive nanogel was highly swollen by the ionization of the PBA moieties, thereby releasing the loaded insulin [49]. Another glucose-responsive polypeptide was developed using a PEG-poly-l-aspartic acid block copolypeptide [50]. 3-aminophenylboronic acid was covalently bonded to the carboxylic acid groups by forming amid bonds [50]. In that study, they prepared two glucose-responsive polymers: PEG-poly(aspartic acid-*co*-aspartiamidophenylboronic acid) (P(Asp-*co*-AspPBA)) and poly(*N*-isopropylacrylamide)-P(Asp-*co*-AspPBA). Using these polymers, complex polymeric micelles (CPMs) for repeated on-off release were formed [50]. The CPMs showed a glucose-responsive sustained insulin release which was proportional to the glucose level and also provided repeated on-off insulin release only at a high glucose concentration [50]. Additionally, the CPMs were capable of protecting insulin from enzymatic attacks because the secondary protein structure of insulin was well maintained even in the presence of trypsin [50].

A PBA moiety with glucose-responsiveness made the hydrophobic segments become water soluble through the binding of glucose [49]. This approach for a phase transition provided controlled insulin release at diabetic glucose levels. Additionally, the PBA with the hydrophobic chain can protect insulin against enzymatic attacks, which provides the glucose-triggered release of intact insulin. Therefore, glucose-responsive systems containing PBA and its derivatives will be a promising platform for controlled and sustained insulin release.

## 7. ATP-Responsive Polypeptides

ATP, a biological energy produced by glucose metabolism, has been recently exploited as a new stimulus trigger because of the abundant ribonucleotides in cells and the concentration gradient between extracellular (<0.4 mM) and intracellular (1–10 mM) conditions [51]. Elevated intracellular ATP levels can enable cargo molecules to be selectively released in the cytoplasm using ATP-responsive delivery carriers. To introduce the ATP-responsive characteristic into the polypeptide, PBA derivatives were attached to the side chain of a polypeptide [52,53].

**Figure 8:**
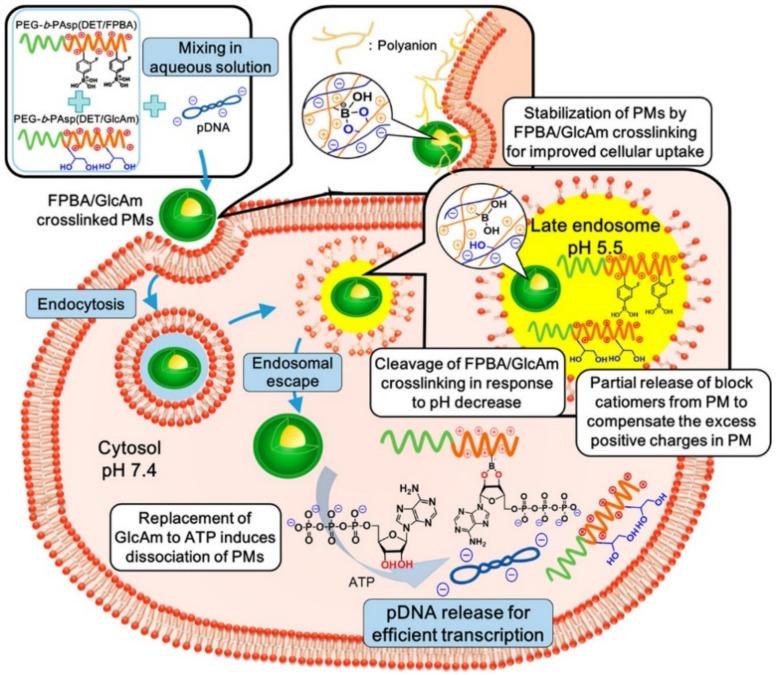
Schematic Illustration of Intracellular Trafficking of FPBA/GlcAm-Cross-Linked Polyplex Micelles (PM), Leading to Smooth Gene Expression, via the Cumulative Processes of Cellular Entry, Endosomal Escape, and ATP-Responsive pDNA Release. Reprinted with permission from [53]. Copyright 2017, American Chemical Society.

Similar to the glucose-responsive systems, ATP consisting of a sugar ring-based structure is capable of strongly binding to PBA, thereby forming a 5- or 6-membered ring [53]. After the complexation, the physical property of the PBA groups becomes hydrophilic via the ionization [53]. To undergo a phase transition at neutral pH, the *p*K_a_ of the PBA derivatives are lowered by the attachment of EWG groups [53]. 4-carboxy-3-fluorophenylboronic acid (FPBA) was attached to the side chain of a polypeptide by the formation of amide bonds (Figure 8) [53]. Its *p*K_a_ value is estimated to be 7.2, at which the sugar molecules bind to the FPBA at pH 7.4 while the complex is broken at a low pH (pH ~ 5.5) (Figure 8) [53]. In that study, they prepared two polypeptides: FPBA-bearing PEG-cationic polypeptide and d-gluconamide-including PEG-cationic polypeptide (Figure 8) [53]. When the two polypeptides were mixed with plasmid DNA in an aqueous phase, the cross-linked polyplex micelles were observed by the formation of the 5- or 6-membered ring structures between the FPBA and gluconamid (Figure 8) [53]. The cross-linked polyplex micelles remarkably captured and protected the pDNA at neutral pH, providing a safe and effective gene delivery in the cytosol (Figure 8) [53]. In contrast, the polyplex micelles were rapidly loosened by the dissociation of the FPBA-gluconamide bonds at late endosomal pH (pH 5.5) [53]. After endosomal escape, the FPBA-bearing cationic polypeptides were inundated with ATP molecules and then re-formed the complex between FPBA and ATP (Figure 8) [53]. As a result, the pDNA molecules were safely and effectively delivered into the cytoplasm [53]. Another study also applied the ATP-responsive characteristic of the FPBA moiety in siRNA delivery systems [52]. The FPBAs were conjugated to the primary amines of a PEG-PLL block copolymer via amidation reaction [52]. The siRNA molecules were condensed by electrostatic attractions and complexation between the FPBA moiety and siRNA, which significantly stabilized the siRNA [52]. When the siRNA-loaded polyplexes were internalized into the cell, the siRNAs were exposed to the cytosol by replacing siRNA with ATP due to the high level of ATP under intracellular conditions [52]. The higher degree of FPBA modification provided the stable formation of the complexes and the ATP-triggered siRNA release at the same time, which enhanced the gene-silencing effect [52].

**Figure 9:**
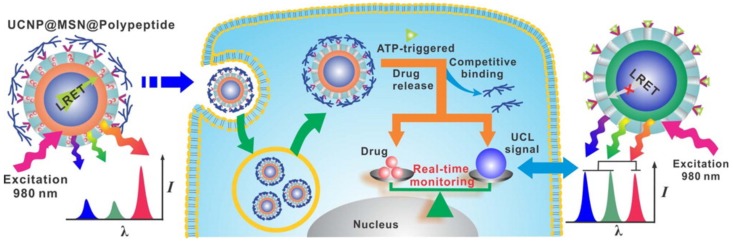
Schematic representation of the real-time monitoring of ATP-responsive drug release. When drugs are released in the cytosol, the fluorescence intensity is much higher. Reprinted permission from [54]. Copyright 2015, American Chemical Society.

Another ATP-responsive method has been recently devised using the interactions between a zinc dipicolylamine analog (TDPA-Zn^2+^) and an ATP molecule (Figure 9) [54]. Branched poly(l-lysine-*co*-l-aspartic acid) used as an ATP-triggered gate keeper was coated onto the surface of mesoporous silica nanoparticles (MSNs) through the coordination bonding of the zinc cations and carboxylates (Figure 9). When the ATP-responsive MSNs are exposed to a high level of ATP, the carboxylates of the branched poly(l-lysine-*co*-l-aspartic acid) are replaced with ATP molecules because the triphosphate anions of ATP have an even higher affinity with TDPA-Zn^2+^, resulting in ATP-triggered drug release (Figure 9) [54]. The ATP-responsive MSNs rarely respond to other anions that could potentially act as a competitive inhibitor, indicating that this system is capable of being activated only in ATP-rich environments [54].

Recently, ATP-responsiveness has been intensively investigated as a new stimulus-switcher [55]. For the past few decades, disulfide bridges have been too widely exploited for controlled intracellular delivery. For this reason, many researchers have endeavored to find a new stimulus trigger for regulated intracellular delivery [55,56,57]. Usually, ATP is highly produced under intracellular environments for cell metabolism and growth, and the concentration difference between extracellular and intracellular conditions is remarkably dispersed, which is quite interesting and useful [51]. Several functional moieties having a strong interaction with ATP molecules were complexed with other molecules that possessed a relatively low affinity [52,53,54]. When they were inundated with ATP molecules, the complex was rapidly deconstructed by replacing the complexing molecules with ATPs, resulting in an ATP-triggered cargo release [52,53,54]. Therefore, the ATP-responsiveness property will be a promising alternative to GSH-triggered systems for intracellular delivery.

## 8. Enzyme-Responsive Polypeptides

Enzyme-responsive properties have been intensively used in drug or gene delivery systems because altered expression profiles of specific enzymes are found at diseased sites [2,58]. For this characteristic, overproduced enzymes are a key stimulus trigger for specific delivery or specific activation of polypeptides [59,60,61]. In this review, the recent studies using prevalent enzymes as a stimulus are covered.

Esterase, a prevalent intracellular enzyme, has been widely used as a stimulus trigger for intracellular delivery [60,62]. Paclitaxel (PTX) molecules are conjugated to PLL via an ester linkage, and then, PTX-conjugated PLL is coated with hyaluronic acid that can target the CD44 receptor overexpressed in cancer cells [63]. When the polyplexes are internalized in the cytosol, the ester bonds are broken by intracellular esterase, resulting in selective PTX release [63]. PTX attached to PLL via an ester bond acts as a pro-drug while PTX evacuated from PLL through the cleavage of the esters starts to show anticancer effects [63].

Matrix metalloproteinase (MMP) is highly over secreted in metastatic cancers, which is a promising differentiation between cancer and normal cells [64,65]. The MMP-cleavable linker is attached between the PEG and PLL blocks, which act as a stealth cell-penetrating agent [39]. When the MMP-cleavable linker is exposed to MMPs, the linkers are dissociated, and the PLL blocks are exposed by the evacuation of PEG [39]. Consequently, the emerging PLL selectively interacts with cancer cell membranes, which give rise to selective drug delivery [39]. A similar approach was found using cathepsin B upregulated in lysosomes or cancer cells as a stimulus trigger [66,67]. The delivery of naked gemcitabine (GEM) into cells is highly difficult due to its high water-affinity. The strategy for GEM delivery is that GEM molecules are conjugated to the polymer chains via cleavable linkers, resulting in satisfactory intracellular delivery as well as the pro-drug at the same time [65,66]. GEM molecules are attached to the PEGylated PLL dendrimer using a cathepsin B-cleavable linker [66]. The GEM-conjugated nanoparticles passively accumulate at the cancer region by relying on an enhanced permeation and retention (EPR) effect [66]. When the nanoparticles are trapped in the lysosomes, the linker is dissociated, and the GEM molecules escape from the PEGylated PLL dendrimers [66]. Additionally, a new protease-cleavable polypeptide was designed by randomly co-polymerizing l-glutamic acid and l-lysine residues [68]. Using PEG-poly(l-glutamic acid-*random*-l-lysine), drug-loaded hydrogels were fabricated by gelation, which could respond to pH, enzyme and temperature. Among them, we focused on a new protease-responsiveness that was easily prepared [68]. Proteases such as MMPs, serine protease, and trypsin overproduced for cancer progression and metastasis degrade amide bonds between l-glutamate and l-lysine residues [68]. Therefore, poly(l-glutamic acid-*random*-l-lysine) is cleaved by protease, thereby deconstructing the hydrogel structures, which is another strategy for protease-activated drug release [68].

A phosphatase-responsive polypeptide was developed as a selective bacteria-killing agent [69]. Phosphoester groups were conjugated to tyrosine residues, which brought about the occurrence of electrostatic attractions within the side chains [69]. The phosphatase-responsive polypeptide had a random-coiled conformation because the level of phosphatase was negligible in normal conditions [69]. In contrast, the phosphatase-responsive polypeptide was transformed to a helical conformation by the cleavage of phosphoester groups that lost an anion-moiety due to the overproduction of phosphatase at bacteria-infected sites [69]. The phosphatase-activated helical formation selectively destabilized bacterial outer membranes only at infection sites showing a high inhibitor effect with minimal side effects [69].

## 9. Conclusions

In this review, we summarized the use of various stimuli-responsive polypeptides and explained the stimuli-responsive mechanisms of the polypeptides based on theoretical backgrounds. The recent progress in stimuli-responsive polypeptides combined with several conjugation methods has been highly promoted in the past decade. Depending on the stimulus, the corresponding functional moiety is conjugated to the side chains or the end of the backbone via the introduction of simple chemistry. Generally, the stimuli-responsive polypeptides provide the desirable functionality at the target region by undergoing physical (phase and conformation) or chemical (dissociation, oxidation, and reduction) transitions. Moreover, polypeptides have an innate biodegradability and biocompatibility unlike other stimuli-responsive polymers. In this review, recent stimuli-responsive polypeptides were explained.

As far as pH-responsive polypeptides are concerned, their conformation-transformable characteristics have been suggested. The conformational transition provides a selective targeting capability as well as therapeutic effects, which will be a promising strategy for improved specificity to diseased sites. The recent polypeptides combined with new stimuli, oxidation and ATP have promising applicability in the major biomedical fields. Considering intracellular conditions, both ROS and ATP levels are significantly elevated, which are strong differentiations that can be used as a new stimulus trigger. The sensitivity of their responsiveness is remarkably higher enabling the selective endowment of functionality at the diseased site.

In conclusion, the recent stimuli-responsive polypeptides have increasingly evolved by applying various strategies to polypeptide-based materials. Many researchers endeavor to impart new and effective functionalities to polypeptides to enhance their practicability. Numerous efforts to develop “new stimuli-responsive polypeptides” will provide promising and important platforms in various biomedical fields.
